# Polystyrene Microplastics and Cadmium Drive the Gut-Liver Axis Through the TLR4/MyD88/NF-κB Signaling Pathway to Cause Toxic Effects on Broilers

**DOI:** 10.3390/toxics13040248

**Published:** 2025-03-27

**Authors:** Ruiwen Fan, Wenqi Tian, Chen Qin, Peng Li, Yuhang Sun, Miao Long, Shuhua Yang

**Affiliations:** College of Veterinary and Animal Science, Shenyang Agricultural University, Shenyang 110866, China; 15321759167@163.com (R.F.); lipeng2018@syau.edu.cn (P.L.); syh2019@syau.edu.cn (Y.S.); longmiao@syau.edu.cn (M.L.)

**Keywords:** polystyrene microplastics, cadmium, broiler chicken, gut-liver axis, inflammation

## Abstract

Nowadays, the risk of oral intake of microplastics (MPs) and cadmium (Cd) by poultry is high. In some industrially polluted areas, the chicken feed samples contain 9.60 × 10^2^ ± 1.09 × 10^2^ MPs per kilogram (mean ± std) in wet weight, and the Cd content in chicken feed has been detected to reach up to 5.61 mg/kg. But scholars still focus more on the toxic effects of MPs and Cd on the liver and intestines of aquatic animals. There are few studies that use poultry as research animals and consider these two organs as an integrated system to investigate the toxicity of MPs and Cd on the gut-liver axis and the mechanisms of inflammation. Therefore, in this research, broilers were used as experimental subjects, and experimental models were established by single or combined exposure of MPs (100 mg/L) and Cd (140 mg/kg) to explore the effects of MPs and Cd on the intestinal mucosae and liver of broilers, as well as the mechanisms behind these toxic effects. In this study, the degree of adverse effects (decreased expression of tight junction proteins, changes in intestinal morphology, abundance and diversity of intestinal flora, liver inflammation) caused by the single exposure group was higher than that of the combined exposure group. The results showed that MPs and Cd induced intestinal damage and liver inflammation in broilers by interfering with the TLR4/MyD88/NF-κB pathway and intestinal flora homeostasis. The toxicity of combined exposure was lower than that of single exposure.

## 1. Introduction

The commercial production of plastics began in the 1940s and grew at an unusually fast rate. According to statistics, plastic production increased from 330 million tons in 2016 to 368 million tons in 2019, and is expected to double in 2039 [1,2]. As plastics are widely used in our daily life such as industries, packaging and pharmaceutical, plastic pollution has become a global concern [3]. The definition of microplastics (MPs) was first proposed in 2004. MPs are plastic particles with a size of less than 5 µm, which can be divided into primary MPs and secondary MPs [4]. Primary MPs are artificial tiny plastic particles used as ingredients in various cosmetics, cleaning products, and biomedical products, such as the exfoliating “beads” added to facial cleansers [5]. Secondary MPs, on the other hand, are formed through the aging, fragmentation, or breakdown of larger plastics due to physical abrasion, ultraviolet (UV) radiation, and heating, such as the abrasion of marine plastic debris caused by wave action and exposure to sunlight [6,7,8]. By migrating between air, water and terrestrial ecosystems, MPs form a complex network of multiple routes. At the same time, most MPs can be released directly or through plastic degradation into the terrestrial environment and accumulate in the soil [9]. The arable soil of southwestern China contain 7100 to 42,960 MPs per kilogram (mean 18,760 per kilogram) [10]. As a result of cultivation and leaching, MPs migrate to deeper soil layers and accumulate in crops such as corn, wheat, and carrots, which can be used as animal feed, may eventually leading to microplastics being eaten by livestock and poultry [11,12,13,14]. After MPs are taken up by See-through Medaka (39.4 nm polystyrene MPs, 10 mg/L, 7 days) and zebrafish larvae (45 μm polystyrene MPs, 1 mg/L, 3 days and polypropylene microfibers, <2 mm size, 2 mg/L, 7 days), they are mainly accumulated in the intestine, MPs are absorbed by intestinal epithelial cells and eventually reach other tissues such as liver, kidney and can even reach the nervous system through the circulatory system [15,16,17]. In the effect of MPs (0.5 μm and 50 μm polystyrene MPs, 1000 μg/L, 5 weeks) exposure on the intestinal flora of mice, it was found that the intestinal flora of mice changed in many aspects of phylum, subject, genus and species after exposure, the intestinal barrier function was impaired, and the intestinal tissue lesions were observed [18]. At the same time, MPs (5 μm polystyrene MPs, 20 mg/kg, 30 days; 5 μm polystyrene MPs, 500 μg/L, 7 days) can lead mice to increased intestinal permeability, allowing more MPs to enter the blood circulation through the intestine and accumulate in the liver, aggravating lipid metabolism disorders, and leading to ROS accumulation and apoptosis of hepatocyte [19,20].

Cadmium (Cd) is a soft silver-white transition metal. It is known to be a poisonous heavy metal which can easily accumulate in the body, posing a serious threat to organisms [21]. Volcanic eruptions, earthquakes and other geological activities with exploitation and smelting of oil fields and mineral resources are the main ways for Cd to appear in the natural environment, and it circulates in nature with the help of water, soil, atmosphere, biology and other media [22]. The Cd content in 75% of agricultural areas in the United States exceeds 0.34 mg/kg, and the Cd content in agricultural soils in Asian countries such as Japan, South Korea, Pakistan, Thailand and India is also high [23,24]. Liver is one of organs where Cd mainly accumulates and exerts toxic effects, studies have shown that Cd (50.2 mg/kg, 30 days; 1.5 mg/kg, 12 weeks) preferentially causes harmful reactions in hepatocytes [25,26]. In the study of Zhang Cong [27], only Cd content in the liver of chickens in the high-dose group (140 mg/kg, 90 days) was significantly higher than control group, while there was no statistical difference between middle-dose group (70 mg/kg, 90 days), low-dose group (35 mg/kg, 90 days) and control group, the degree of inflammatory injury and toxic effects caused by Cd on the liver increased in a dose-dependent manner. At the same time, Cd can damage intestinal tissue, studies have shown that Cd exposure (5 to 100 mg/L) induced a decrease in the expression level of intestinal tight junction protein genes in mice, a significant change in bacterial population and relative abundance, and a significant decrease in probiotics, especially the decrease in the ratio of the relative content of Firmicutes and Bacteroides [28,29,30].

According to soil pollution status report of China, Cd ranks first among farmland metal pollutants, exceeding soil environmental quality standards of China [31]. High concentrations of Cd have been reported in nine coastal rivers in China [32]. Because MPs can migrate in farmland and aquatic environments, and studies have clarified the adsorption of heavy metal Cd by MPS in the environment [33], MPs and Cd are likely to appear in the same environment at the same time. Cd, as a plastic additive, is often detected in various plastic products [34]. Studies have shown that MPs are present in poultry manure in livestock and poultry farms in southern China, and their transmission channels may spread MPs to chickens through faucets, bowls, and feed bags [35]. And recent studies found that MPs and heavy metals which attached to MPs surfaces can be absorbed by organisms [36,37]. MPs intake provides a way for Cd to be transferred to organisms. The results showed that exposure of grass carp to MPs (30 and 300 μg/L) or Cd (10 and 20 mg/L) alone for 96 h induced severe oxidative stress, meanwhile higher MPs concentration led to more Cd accumulation in gills, intestines, liver and kidneys [38]. After 10 days exposure to zebrafish with 5 μm MPs (100 μg/L) and Cd, it was found that MPs could significantly increase the accumulation of low concentration Cd (15 μg/L) in intestine, gill, skin and muscle tissues, while reducing the accumulation of high concentration Cd (150 μg/L) in liver, intestine, gill and muscle tissues [39]. At present, the research on the combined toxic effects of MPs and Cd is mainly limited to aquatic animals and invertebrates. Studies on the effects of MPs and Cd exposure on poultry and their potential toxicity are still limited. It is necessary to further determine the toxic effects of MPs and Cd co-exposure on poultry.

Therefore, the purpose of this study is to analyze the effects of MPs and Cd on the intestine and liver of broilers and the mechanism of toxic effects, and to provide a theoretical basis for exploring the toxic mechanism of MPs and Cd on poultry.

## 2. Materials and Methods

### 2.1. Materials

Fluorescent polystyrene MPs with a diameter of 0.1 microns were purchased from Jiangsu Zhichuan Co., Ltd. (Nantong, China). Cadmium chloride was purchased from Sinopharm Chemical Reagents Co., Ltd. (Shanghai, China). The antibodies used for Western blot analysis were as follows: TLR4 (YN5450, Rabit, 1:1000), MyD88 (YT2928, Rabit, 1:1000), NF-κB (YM8001, Rabit, 1:1000), Occludin (YN2865, Rabit, 1:1000), Claudin-1 (YT0942, Rabit, 1:1000), ZO-1 (YN1410, Rabit, 1:1000) and β-actin (YM8010, Rabit, 1:1000) all purchased from ImmunoWay (Plano, CA, USA). IgG-HRP secondary antibody was purchased from ImmunoWay (Plano, CA, USA).

### 2.2. Animal Research

All animal experiments were carried out in strict accordance with the “Animal Model Planning Law of the People’s Republic of China”, and all processes were carried out in accordance with the Ethical Committee of Shenyang Agricultural University and the approved principles of laboratory veterinary medicine (No. 201806014). Before the experiments, the cages, water tanks, feeing slots and sites of the chicken house were disinfected. After 48 h of ventilation, the experimental animals were led in and raised in stainless steel cages (five chicks/cage). During the experiment, they were free to drink and feed throughout the day, and kept in a controlled room (temperature: 33 °C for the first 7 days to adapt to the environment, decreased by 1 °C every 3 days, and was finally controlled at 22 °C; relative humidity: kept at 65% in the first 7 days, then decreased 5% every week, and finally keep at 55%; lighting: 24 h lighting, 10 lux). Broiler chicks were fed with the basal diet based on the full-price compound feed of broiler chicks to meet the nutritional needs of primary broiler chicks.

Sixty 1-day-old healthy white feather broilers (40 ± 2 g) were bought from Shenyang huamei livestock and poultry breeding base and randomly divided into four groups (*n* = 15) after one week of adaptive feeding: control group (given normal drinking water and basic feed), MPs group (given 100 mg/L MPs drinking water and basic feed, new contaminated water was changed daily), Cd group (given 140 mg/kg Cd mixed feed and normal drinking water) and MPs + Cd group (given 100 mg/L MPs drinking water and 140 mg/kg Cd mixed feed). The dosage of Cd referred to the study of oral median lethal dose of Cd to chickens (LD50 = 218.44 mg/kg·B·W) [40] and the study of Zhang Cong (the dose of Cd was 140 mg/kg (3/5 LD50)) [27]. The amount of MPs referred to Hou LL’s research, because chickens are animals with high metabolic rates and long lifespans which are highly adaptable to poisons, this study used a high dose of MPs (100 mg/L) for research [41]. All groups were free to eat and drink water, as well as the body weight and health status were recorded daily. When the experiment was carried out for 21 days, the broilers were killed after fasting and water deprivation for 12 h after the last administration. And the ileum, cecal contents and liver were collected for subsequent experiments. The liver weight was weighed to measure the liver index.

Liver index = (liver mass/body mass) × 100%.

### 2.3. Histopathology

#### 2.3.1. HE Staining

The tissue was fixed with 4% paraformaldehyde solution (Sinopharm Chemical Reagents Co., Ltd., Shanghai, China), then embedded in paraffin and cut into 5 μm thick sections, washed with gradient ethanol, dewaxed with xylene for 2 h, and stained with hematoxylin (Merck, Darmstadt, Germany) and eosin (Sigma Aldrich, St. Louis, MO, USA). The liver tissue sections were dehydrated with gradient alcohol and xylene, and then sealed with neutral resin. The slices were examined by optical microscope (Leica DM750 microscope, Leica, Beijing, China) and photographed. The villus length and crypt depth of ileum were measured by ImageJ 1.54g Software, also it was used for semi-quantitative analysis of the results.

#### 2.3.2. DAPI Staining

Ileal tissue was embedded in OCT embedding agent. The 10 µm thick slices were prepared, rewarmed at room temperature and dried. Then, the slices were put into PBS solution to remove the fixative after histochemical stroke circles, and the DAPI dye solution (Shanghai Biyuntian Biotechnology Co., Ltd., Shanghai, China) was added. After incubation in the dark, the slices were washed again and sealed with an anti-fluorescence quenching sealing agent. Finally, the images were obtained by confocal laser scanning microscope (the excitation wavelength of green fluorescence was 488 nm). ImageJ was used for semi-quantitative analysis of the results.

### 2.4. Biochemical Analysis

According to the manufacturer’s instructions, the level of ileal secretory immunoglobulin A (sIgA) was determined by ELISA kit (MM-049302, Shanghai Enzyme Immunobiotechnology Co., Ltd., Shanghai, China). The levels of cytokines IL-1β (ml059835), IL-2 (ml059836), IL-4 (ml059838), IL-6 (ml059839), IL-10 (ml059830) and TNF-α (ml002790) in tissues were measured by ELISA kit (Shanghai Enzyme-linked Biotechnology Co., Ltd., Shanghai, China). The levels of LPS in tissues were measured by ELISA kit (JYM0109Ch, Wuhan Colorful Gene Biotech Co., Ltd., Wuhan, China). At least three biological replicates and three technical replicates were performed on each group of data. The testing procedure was carried out according to each instruction provided by the company above (all the loading amounts of samples to be tested were 10 μL, then diluted five times). The linear regression curve was drawn for the standard samples given by each ELISA kit, and the concentration value of each sample was calculated according to the standard curve.

### 2.5. Analysis of Target Gene Expression

RNA was extracted from chicken tissues by using RNA extraction kits (RM201, Nanjing Noviacan Biotechnology Co., Ltd., Nanjing, China). After detecting RNA concentration and quality, the extracted RNA was reverse transcribed by qRT-PCR kit (R223-01, Nanjing Nuoweizan Biotechnology Co., Ltd., Nanjing, China) to obtain cDNA and remove gDNA. The specific primers were presented in Table 1. And quantitative analysis was conducted by SYBR green real-time fluorescent quantitative PCR. The qRT-PCR system: 2 × SYBRGREEN MIX 10 μL (Q711-02, Nanjing Noviacan Biotechnology Co., Ltd., Nanjing, China), each 0.4 μL upstream and downstream primer (10 μmol·L^−1^), cDNA template 2.0 μL, ddH2O 6.2 μL. The mRNA expression level was calculated by 2^−△△Ct^.

### 2.6. Western Blotting of Protein Expression

Total protein was extracted from tissues using a protein extraction kit (BC3790, Beijing Solarbio Science & Technology Co., Ltd., Beijing, China). The protein concentration in tissue lysates was measured using a BCA kit (ZJ102, Shanghai Yamei Biotechnology Co., Ltd., Shanghai, China). An amount of 20 μL of proteins were loaded onto SDS-PAGE gel (Shanghai Yamei Biotechnology Co., Ltd., Shanghai, China). The sample was transferred to a PVDF membrane, then blocked for 2 h using a 5% skimmed milk powder solution. The primary antibody was incubated overnight at a controlled temperature of 4 °C. The PVDF membrane was washed four times with TBST before adding the secondary antibody, then incubated at room temperature for 2 h. The sample was washed four times with TBST again. After observing the results of protein development, ImageJ software was used for semi-quantitative analysis of the results and to analyze the grayscale values of protein bands.

### 2.7. 16s rDNA Gene Sequencing and Analysis

The total genomic DNA was extracted by CTAB/SDS method. The DNA concentration and purity were detected on 1% agarose gel, and the V3–V4 region of bacterial 16S rDNA was amplified by PCR. After PCR amplification, the PCR products were detected by electrophoresis with 1.8% agarose. The PCR product of the target region (10 μL system) was mixed with VAHTSTM DNA Clean Beads magnetic beads according to 1:1, and the magnetic beads were used to screen the fragments. After Solexa PCR, it was detected by electrophoresis, and then quantitatively analyzed by ImageJ. Finally, high-throughput sequencing was performed. The data were filtered by Trimmomatic v0.33 software and cutadapt 1.9.1 software quality control, and denoised by QIIME2 2020.6 to obtain the sequence that removed the chimera. Eventually, in order to describe the composition of microorganisms in the jejunum contents of broilers, the obtained OTUs were classified according to the different levels of phylum, class, order and species.

### 2.8. Statistical Analysis of Data

Statistical analysis was performed by using IBM SPSS Statistics 25 software (SPSS Inc., Chicago, IL, USA), and the results were presented as mean ± standard deviation (Mean ± SD). In order to compare the differences between different experimental groups (control group, MPs group, Cd group and MPs + Cd group), the data were normalized. Then, the one-way ANOVA method and Tukey’s multiple comparison tests were conducted. GraphPad Prism 8 software (GraphPad Software, La Jolla, CA, USA) is used to draw graphs. *p* < 0.05 indicated significant difference, while *p* < 0.01 indicated extremely significant difference. So when the results showed *p* < 0.05, these differences can be considered statistically significant.

## 3. Results and Analysis

### 3.1. Combined Exposure of MPs and Cd on the Body Damage of Broilers

**Figure 1:**
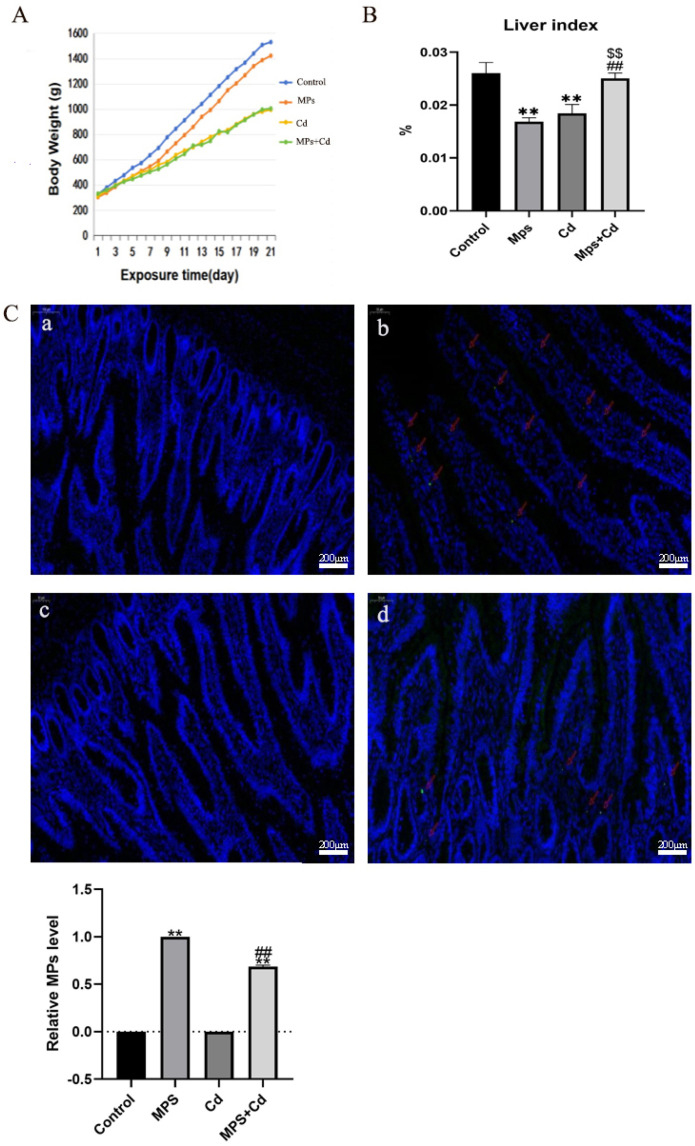
Combined exposure of MPs and Cd on broiler body damage. (**A**): Broiler weight growth changes. (**B**): broiler liver index ratio. (**C**): Broiler ileum tissue frozen section enlarged 50 times. Blue fluorescence is stained with DAPI. Red arrow: MPs fluorescent green spots. (**a**): control group; (**b**): MPs group; (**c**): Cd group; (**d**): MPs + Cd group. **: compared with control group, ##: compared with MPs group, $$: compared with Cd group. **, ##, and $$ indicated that there was a very significant difference in comparison (*p* < 0.01).

In order to explore the effects of MPs and Cd separately or in combination on the body of broilers, we recorded body weight changes, liver index ratios and intestinal DAPI staining of broilers. Compared with the control group, the body weight and liver index of MPs group, Cd group and combined exposure group decreased to varying degrees (Figure 1A,B). Under the condition of the same daily feed intake in broilers, although the body weight of the combined exposure group was significantly lower than that of the MPs group, there was no significant difference compared with the Cd group, and even the combined exposure group almost reversed the results of the single exposure group to the normal level in the liver index. The results showed that MPs and Cd had a negative regulatory effect on the growth of broilers, and the effect of MPs combined with Cd on body weight and liver index of broilers was reduced. The distribution of MPs in the intestinal tissue was shown by DAPI staining (Figure 1C). Fluorescent green spots (MPs) were observed in the frozen sections of the ileum tissues of broilers in the MPs group and the MPs + Cd group. It indicated that fluorescent MPs entered the intestinal tissue of broilers.

### 3.2. Effects of Combined Exposure of MPs and Cd on Intestinal Morphology and Barrier Function in Broilers

**Figure 2:**
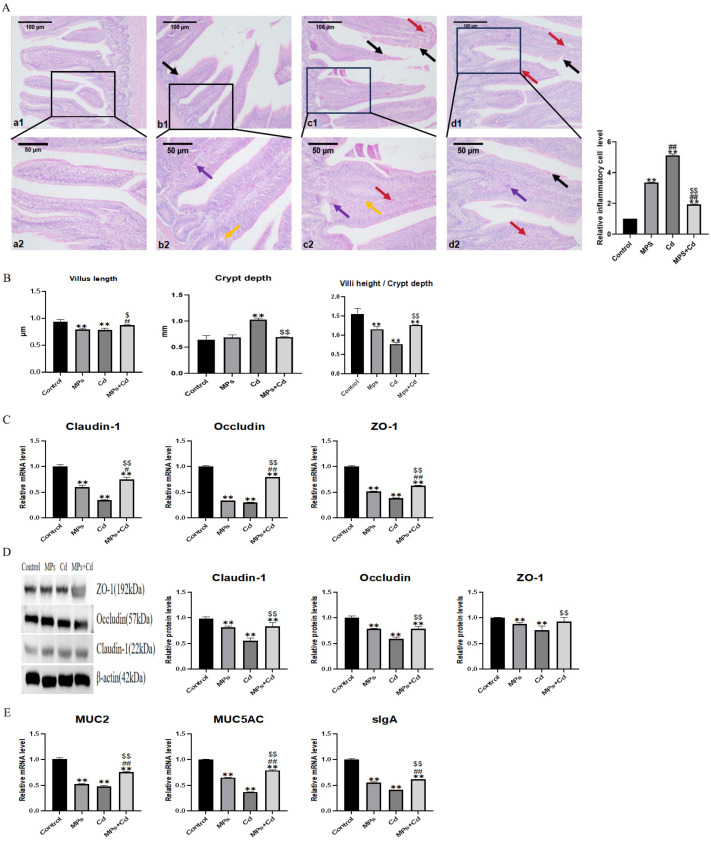
Regulatory effects of combined exposure of MPs and Cd on intestinal morphology and mucosal function of broilers. (**A**): HE staining results of broiler ileum tissue. (**B**): Effects of MPs and Cd on ileal morphology of broilers. (**a1**–**d1**): ileal structure in 100-fold visual field, (**a2**–**d2**): ileal structure in 200-fold visual field. (**a1**,**a2**): control group, (**b1**,**b2**): MPs group, (**c1**,**c2**): Cd group, (**d1**,**d2**): combined exposure group. Black arrow: fluff fracture. Red arrow: bleeding. Purple arrow: Inflammatory cell infiltration. Yellow arrow: vacuolization. (**C**): Effects of MPs and Cd on the mRNA of Claudin-1, Occludin and ZO-1 in broilers. (**D**): Effects of MPs and Cd on the relative content of Claudin-1, Occludin and ZO-1 protein (20 μL loading amount) in broilers. (**E**): Effects of MPs and Cd on the mRNA expression of MUC2, MUC5AC and sIgA. Values are expressed as AVG ± SD. **: compared with control group, # and ##: compared with MPs group, $ and $$: compared with Cd group. # and $ indicated significant difference in comparison (*p* < 0.05). **, ##, and $$ indicated that there was a very significant difference in comparison (*p* < 0.01).

In order to detect the effect of MPs and Cd on intestinal morphology, we performed HE staining on the ileum tissue of broilers (Figure 2A). Compared with the control group, the ileum villi of broilers in the MPs group showed slight mucosa vacuolization, inflammatory cell infiltration and villus epithelial shedding. The ileum injury in the Cd group was more serious than that in the MPs group, which was characterized by villus rupture, congestion and hemorrhage, a large number of inflammatory cell infiltration and epithelial cell shedding. Compared with the Cd group, the ileal villus of the combined exposure group was less broken, and the inflammatory cell infiltration and epithelial cell shedding were alleviated. It is suggested that MPs and Cd can cause damage to the intestinal tract of broilers, and the damage is reduced after combined exposure. We further found that, compared with the control group, the ileal villus length of the MPs group and the Cd group decreased significantly (*p* < 0.01), while the combined exposure group could restore it to the control group level, and the ratio of intestinal villus length to crypt depth also showed this trend (Figure 2B). Compared with the control group, the intestinal crypt depth of the Cd group increased significantly (*p* < 0.01). But there was no significant change in the intestinal crypt depth of the MPs group and the combined exposure group. The results showed that MPs and Cd could damage the ileal villi of broilers, and Cd could also damage the ileal crypts of broilers. The combination of MPs and Cd could reduce these damages.

We examined the protein abundance and mRNA expression levels of tight junction proteins Claudin-1, Occludin and ZO-1 that maintain the intestinal mucosal barrier. Compared with the control group, the gene and protein expression levels of tight junction proteins in MPs group and Cd group decreased significantly (*p* < 0.01) (Figure 2C,D). The combined exposure group significantly reversed the down-regulation of tight junction protein abundance induced by MPs or Cd single exposure. It shows that MPs and Cd can damage the physical barrier of the ileum of broilers, and the damage is alleviated after combined exposure.

In order to study the mechanism of MPs and Cd on the intestinal chemical barrier, we detected the gene expression levels of ileal mucin (MUC2, MUC5AC) and secretory globulin A (sIgA). The expression of MUC2, MUC5AC and sIgA genes in the MPs group and the Cd group was significantly lower than that in the control group (*p* < 0.01), and the expression level of the combined exposure group was significantly higher than that of the MPs group and the Cd group (*p* < 0.01) (Figure 2E).

The above results showed that both MPs and Cd could damage the intestinal morphology and barrier function of broilers, but the damage was alleviated after the combination of MPs and Cd.

### 3.3. Effects of Combined Exposure of MPs and Cd on Intestinal Inflammation in Broilers

**Figure 3:**
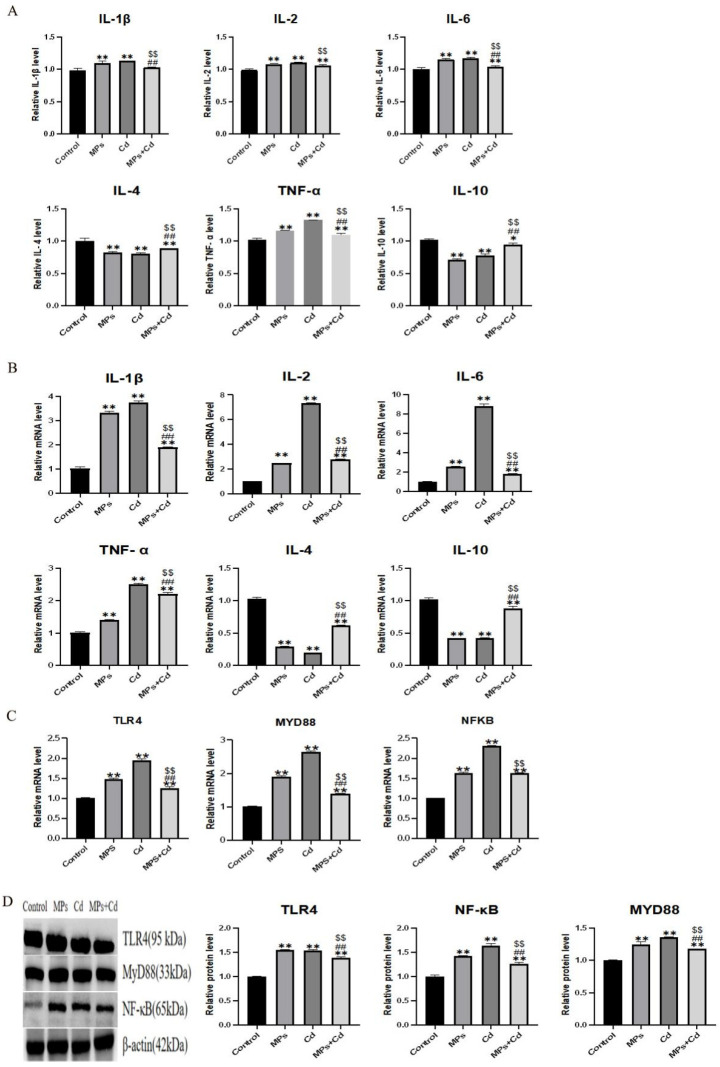
Effects of combined exposure of MPs and Cd on the regulation of intestinal inflammation in broilers. (**A**): Effects of MPs and Cd on the content of inflammatory factors in the ileum of broilers (10 μL loading amount of sample). (**B**): Effects of MPs and Cd on the relative mRNA content of ileum inflammation in broilers. (**C**): Effects of MPs and Cd on the relative mRNA content of the TLR4/MyD88/NF-κB inflammatory pathway in ileum of broilers. (**D**): Effects of MPs and Cd on key protein content of the TLR4/MyD88/NF-κB inflammatory pathway in ileum of broilers (20 μL loading amount). Values are expressed as AVG ± SD. * and **: compared with control group, ##: compared with MPs group, $$: compared with Cd group. * indicated significant difference in comparison (*p* < 0.05). **, ##, and $$ indicated that there was a very significant difference in comparison (*p* < 0.01).

When the intestinal barrier is damaged, the intestinal permeability increases, and the invasion of pathogens or exogenous stimuli can cause tissue inflammation. In order to study the effect of combined exposure of MPs and Cd on intestinal inflammation, we detected the content and mRNA expression of pro-inflammatory factors IL-1β, IL-2, IL-6 and TNF-α, anti-inflammatory factors IL-4 and IL-10 in ileum of broilers. Although the content and mRNA expression of pro-inflammatory factors IL-2, IL-6 and TNF-α in MPs group, Cd group and combined exposure group were significantly increased compared with the control group (*p* < 0.01), the content and mRNA expression of pro-inflammatory factors IL-1β, IL-6 and TNF-α in the combined group were significantly lower than those in the two single exposure groups (*p* < 0.01) (Figure 3A,B). The content and mRNA expression of anti-inflammatory factors IL-4 and IL-10 in each group showed an opposite trend compared with pro-inflammatory factors. It shows that both MPs and Cd can cause inflammation in the ileum of broilers, thus destroying the intestinal mucosal immune barrier and causing intestinal damage, but the combined exposure of the two can reduce this damage.

Changes in mRNA and protein abundance based on the TLR4/MyD88/NF-κB inflammatory pathway also support this view. Compared with the control group, the relative mRNA and protein contents of TLR4, MyD88 and NF-κB in ileum of broilers in MPs, Cd and combined exposure groups were increased (*p* < 0.01) (Figure 3C,D). Compared with MPs group and Cd group, mRNA and protein relative contents of TLR4, MyD88 and NF-κB in combined exposure group were decreased (*p* < 0.01).

These results suggest that MPs and Cd can cause inflammatory responses in the ileum of broilers and regulate the TLR4/MyD88/NF-κB pathway, but combined exposure can reduce these damages.

### 3.4. Regulation of Combined Exposure of MPs and Cd on Cecal Microflora in Broilers

**Figure 4:**
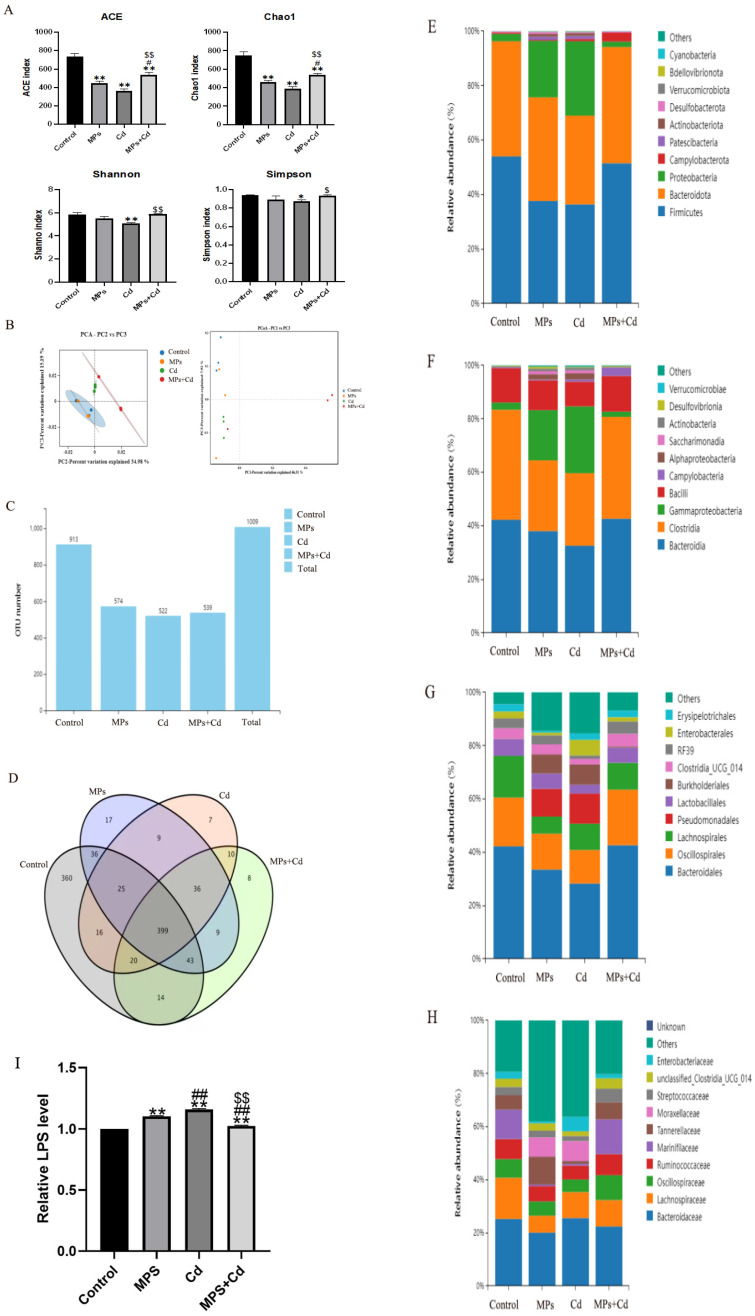
Regulation of cecal microbiota in broilers by combined exposure to MPs and Cd. (**A**): Alpha Diversity Index. (**B**): Analysis of the β diversity of the jejunal contents in broiler chickens. (**C**): Number of OTUs. (**D**): Venn diagram. (**E**): Analysis of OUT phylum levels in the cecal contents of broilers. (**F**): Analysis of OUT class levels of cecal contents in broilers. (**G**): Analysis of OUT order levels of cecal contents of broilers. (**H**): Analysis of OTU species levels of cecal contents of broilers. (**I**): Effects of MPs and Cd on the content of LPS in ileum of broilers (10 μL loading amount of sample). Values are expressed as AVG ± SD. * and **: compared with control group, # and ##: compared with MPs group, $ and $$: compared with Cd group. *, #, and $ indicated significant difference in comparison (*p* < 0.05). **, ##, and $$ indicated that there was a very significant difference in comparison (*p* < 0.01).

In order to further study the regulation of intestinal microbiota by combined exposure of MPs and Cd, the cecal microbiota was analyzed by 16sr DNA sequencing. The α diversity of the flora was reflected by Chao1, ACE, Shannon and Simpson indexes. It can be seen that although compared with the control group, the Chao1 and ACE indexes of the cecal flora of broilers after single or combined exposure were significantly lower (*p* < 0.01), but the combined exposure group was still significantly higher than the single exposure group (*p* < 0.01) (Figure 4A). Compared with the Cd group, the Shannon index (*p* < 0.01) and Simpson index of the combined exposure group were significantly increased (*p* < 0.05). It shows that the richness of intestinal flora and the diversity of community in the combined group are higher than those in the single exposure group. The β diversity of the microbiota was shown that samples in each group were close and the differences within the group were small (Figure 4B). PCA and PCoA points were separated between the control group and the exposure group, indicating that the microbial diversity of the cecal content of broilers was affected by MPs and Cd.

We further performed high-throughput sequencing of cecal contents of broilers. After OTU/ASV analysis, the total number of OTUs was 1009. The control group, MPs group, Cd group and combined exposure group obtained 913, 574, 522 and 539 OTUs on average, respectively (Figure 4C). It can be seen that the number of unique OTUs in the control group, MPs group, Cd group and combined exposure group was 360, 17, 7 and 8, respectively (Figure 4D). Among the common OTUs, compared with the common OTU number between the Cd group and the control group, the common OTU number between the combined exposure group and the control group was more.

We analyzed the changes in the relative abundance composition of cecal microflora in broilers from the levels of phylum, class, order and species. Compared with the control group, the relative abundance of Firmicutes, Bacteroidetes, Bacteroidia, Clostridia, Bacteroidales, Oscillospirales, Lachnospirale and Ruminococcaceae in cecal flora of broilers in MPs group and Cd group decreased, as well as the richness of Proteobacteria, Clostridia, Gammaproteobacteria and Pseudomonadales increased significantly, while the changes in the combined exposure group were not significant compared with the single exposure group (Figure 4E–H). After that, we detected the main component of the cell wall of Gram-negative bacteria in the intestine of broilers—LPS. The results showed that the LPS levels in the MPs and Cd groups were significantly higher than those in the control group, while the LPS in the combined exposure group was lower than that in the single exposure group (Figure 4I).

The above results show that the exposure of MPs and Cd can cause changes in the abundance of intestinal flora. The exposure of MPs and Cd will cause changes in the abundance of intestinal flora, resulting in a decrease in beneficial bacteria and an increase in potential pathogenic bacteria, destroying the intestinal microecological balance and weakening the intestinal barrier function. At the same time, the increase in LPS in the intestine leads to inflammatory response. But the combined exposure will reduce this change.

### 3.5. Regulation of Combined Exposure of MPs and Cd on Liver Inflammation in Broilers

In recent years, the study of intestinal-derived liver inflammation has received continuous attention, and the relationship between intestinal microbiota and liver inflammation has gradually emerged. In order to study the relationship between intestinal flora disorders and hepatitis after changes in intestinal permeability, we also analyzed the effects of MPs and Cd exposure on liver inflammation in chickens.

**Figure 5:**
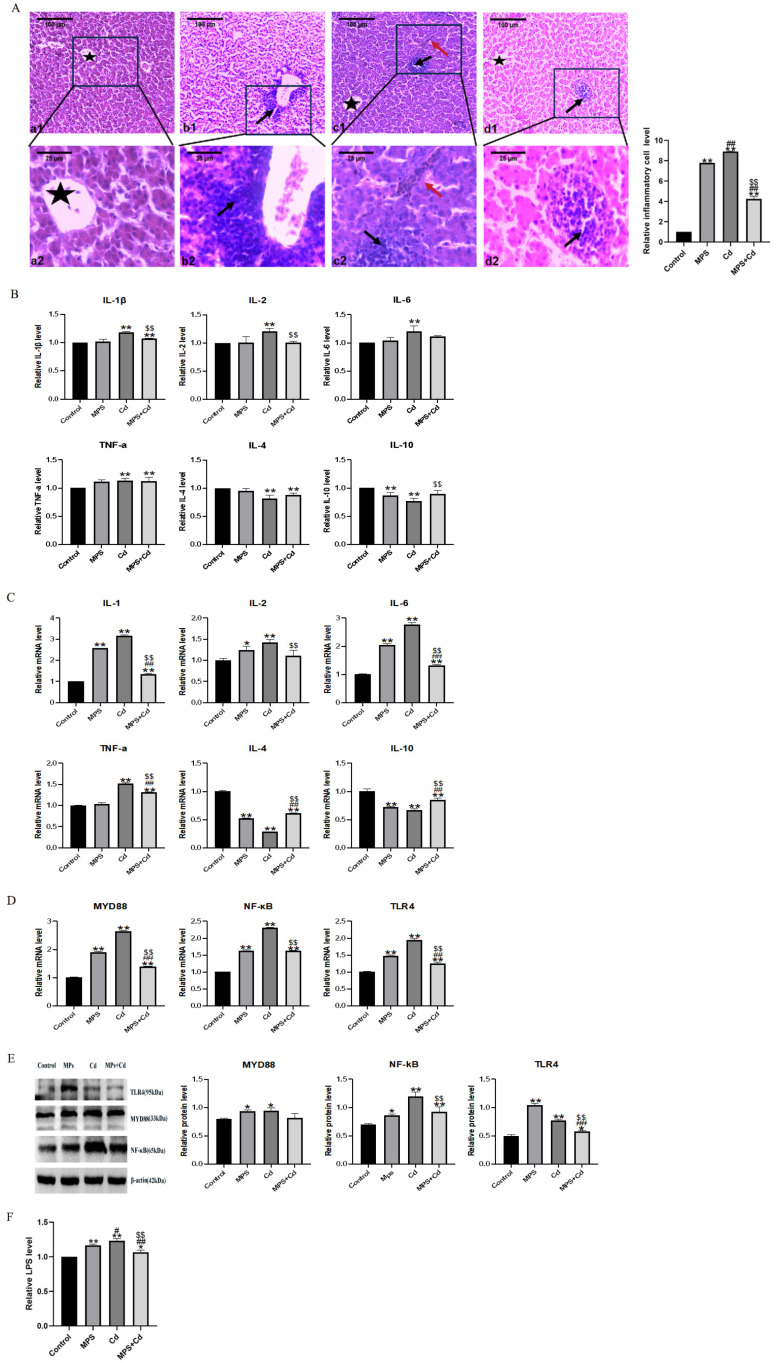
Regulation effect of combined exposure of MPs and Cd on liver inflammation in broilers. (**A**): HE staining results of broiler liver tissue. (**a1**–**d1**): Ileum structure in 100 times visual field, (**a2**–**d2**): Ileum structure in 400 times visual field. (**a1**,**a2**): control group, (**b1**,**b2**): MPs group, (**c1**,**c2**): Cd group, (**d1**,**d2**): combined exposure group. Black arrow: Inflammatory cell infiltration. Red arrow: bleeding. Five-pointed star: central vein. (**B**): Effects of MPs and Cd on the content of inflammatory factors in liver of broilers (10 μL loading amount of sample). (**C**): Effects of MPs and Cd on relative mRNA expression of liver inflammation in broilers. (**D**): Effects of MPs and Cd on mRNA content of the TLR4/MyD88/NF-κB inflammatory pathway in ileum of broilers. (**E**): Effects of MPs and Cd on the content of key proteins in the TLR4/MyD88/NF-κB inflammatory pathway in ileum of broilers (20 μL loading amount). (**F**): Effects of MPs and Cd on the content of LPS in liver of broilers (10 μL loading amount). Values are expressed as AVG ± SD. * and **: compared with control group, # and ##: compared with MPs group, $$: compared with Cd group. * and # indicated significant difference in comparison (*p* < 0.05). **, ## and $$ indicated that there was a very significant difference in comparison (*p* < 0.01).

In order to analyze the liver toxicity and regulatory mechanism of combined exposure of MPs and Cd on broiler chickens, we observed liver pathological sections. The control group showed dense and tight liver tissue, once the liver tissue of the MPs group, the Cd group and the combined group was loose, and inflammatory infiltration was observed (Figure 5A). The Cd group was the worst and even had bleeding. Compared with the single exposure group, the combined group was significantly improved. The content and mRNA expression of pro-inflammatory cytokines IL-1β, IL-2, IL-6 and TNF-α, anti-inflammatory cytokines IL-4 and IL-10 in the liver were detected. The combined exposure group reversed the increase in pro-inflammatory cytokines IL-1β, IL-2, IL-6 and TNF-α and their mRNA expression caused by MPs group and Cd group, as well as reserved the decrease in anti-inflammatory cytokines IL-4 and IL-10 content and their mRNA expression (Figure 5B,C). Further validation was performed based on the TLR4/MyD88/NF-κB pathway. The mRNA expression levels of TLR4, MyD88, and NF-κB were analyzed by qRT-PCR test (Figure 5D). The mRNA expression levels of the three genes were significantly decreased after the addition of MPs or Cd compared with the control group (*p* < 0.01), and the results were reversed in the combined exposure group (*p* < 0.01). The results of Western blot showed the same trend as qRT-PCR results (Figure 5E). We detected the content of LPS in liver. The results showed that the LPS levels in the MPs and Cd groups were significantly higher than those in the control group, while the LPS in the combined exposure group was lower than that in the single exposure group (Figure 5F).

The above results indicate that the mechanism of MPs or Cd single exposure causing liver inflammation in broilers is due to the inflammatory response caused by LPS in the intestinal flora, but the combined exposure can reduce liver inflammation.

## 4. Discussion

With the increasing pollution of plastic waste, MPs have become one of the most widespread pollutants in the world. Most of the previous studies on MPs were aquatic organisms (such as fish, shrimp, algae) [42]. There are few articles on birds living at waterside. At the same time, more and more evidence show that MPs pollution exists in livestock and poultry farms. In a previous study, MPs residues were detected in the intestine, liver and skeletal muscle of chickens in Chinese chicken farms [43]. At the same time, due to the pollution of mineral supplements, some limestone added to chicken feed may contain relatively high levels of Cd [44]. Therefore, in this study, chickens were used as experimental subjects to explore the toxic effects of polystyrene MPs and Cd. The results showed that the mechanism of the above toxicity was related to the inflammatory response and the change in intestinal flora.

Polystyrene MPs and Cd can damage the intestinal tract. The results of this experiment showed that polystyrene MPs or Cd could separately cause intestinal damage such as intestinal villus damage and shortening, intestinal wall thinning. The protein abundance, mRNA expression levels of tight junction proteins (Claudin-1, Occludin and ZO-1), the gene expression of ileal mucin (MUC2, MUC5AC) and secretory globulin A (sIgA) decreased. Studies have shown that after exposure to 500 μg/L Cd in a tank for 14 days, the intestinal histology of carp was diseased [45]. Mice were also exposed to polystyrene MPs (5 μm, 100 and 1000 μg/L, 6 weeks), the gene levels of Muc1, Muc2, and Muc3 in the intestine were significantly decreased [46]. These are consistent with the results of this study. These results indicated that polystyrene MPs and Cd could damage the intestinal tract of broilers.

To more accurately explain how polystyrene MPs and Cd induce inflammation in the gut-liver axis of broilers, we compared the MPs group and the Cd group with the control group. We found that both of them can cause intestinal flora disorder in broiler intestine. Polystyrene MPs and Cd reduced the proportions of Firmicutes and Bacteroidetes, and beneficial bacteria such as Lachnospiraceae, Oscillospiraceae, and Ruminococcaceae which produce butyrate and other short-chain fatty acids also decreased after exposure to polystyrene MPs and Cd. This is consistent with the findings of Wei W [47], the relative abundance of Proteobacteria and Bacteroidetes increased significantly, and the abundance of Actinobacteria, Firmicutes and Fusobacteria decreased significantly after treatment with polystyrene MPs (1 mg/L, 21 days) and Cd (5 mg/L, 21 days), respectively. These results indicate that both polystyrene MPs and Cd can alter the composition of the intestinal microbiota in broilers, particularly the proportion of Gram-negative bacteria. Therefore, it is important to study how polystyrene MPs and Cd affect the mechanism of Gram-negative bacteria.

LPS is a key component of the cell wall of Gram-negative bacteria. After treating broilers with polystyrene MPs and Cd separately, the LPS content in the intestines and liver significantly increased. Toll-like receptor 4 (TLR4) serves as a link between immune responses and gut microbiota, upon activation, the signaling adaptor protein myeloid differentiation factor 88 (MyD88) is recruited and binds to the cytoplasmic domain of TLR4, releasing downstream nuclear factor-kappa B (NF-κB), which leads to the release of pro-inflammatory factors and triggers an inflammatory response [48]. Therefore, this study selected the TLR4/MyD88/NF-κB signaling pathway to study the inflammatory response of polystyrene MPs and Cd to the intestine and liver of broilers. LPS can act as an agonist of TLR4 signaling pathway and ultimately activate NF-κB signaling pathway [49,50]. In this experiment, the exposure of these two toxic substances separately increased the levels of pro-inflammatory factors in the intestine and liver, decreased the levels of anti-inflammatory factors, and decreased the expression of key genes and proteins in the TLR4/MyD88/NF-κB inflammatory pathway. LPS plays an important role in mediating intestinal inflammation in inflammatory bowel disease, which can induce increased permeability of intestinal epithelial tight junctions [51]. To some extent, increased intestinal permeability means an increased risk of intestinal microbial exudation. Studies have shown that intestinal bacterial LPS can induce liver inflammation in ducks [52]. This further explains the possible reason for the inflammation of polystyrene MPs and Cd in the gut-liver axis of broilers in this study is that the two toxins increase the LPS level in the intestine and increase the intestinal permeability, causing inflammation to spread to the liver.

It is very interesting that all the above damage to the intestine and liver of broilers in this study, the single exposure of polystyrene MPs or Cd caused greater damage, and the combined exposure of the two resulted in reduced damage. These are similar with the results of another research on Oryzias melastigma, polystyrene MPs treatment reduced the diversity and abundance of intestinal flora, while the combined treatment of polystyrene MPs (2.5 μm, 100 μg/L, 1 month) and Cd (100 mg/L, 1 month) increased them [53]. The possible reason is due to the adsorption of polystyrene MPs, so that Cd is adsorbed on polystyrene MPs, and this toxicity antagonism is affected by many factors. In the study of a variety of aquatic animals, the combined toxicity of these two toxins is related to the exposure concentration of Cd, the size and concentration of polystyrene MPs particles [39,54,55]. After 5 days of exposure to polystyrene MPs, the accumulation of polystyrene MPs particles in the intestine of tilapia reached the peak, because the smaller plastic particles were more likely to remain in the intestinal tissue, while the larger particles (5 μm) would be excreted through the feces [56]. At the same time, the adsorption capacity of polystyrene MPs for Cd is affected by temperature and pH value, higher temperature and pH value and lower salinity contribute to the adsorption of Cd by polystyrene MPs [57,58]. In the simulated gastrointestinal environment, it was also found that the adsorption of heavy metal Cd by polystyrene MPs increased [59]. In this experiment, due to the adsorption of polystyrene MPs, the particle diameter increased after combined exposure with Cd, which was more likely to be discharged with feces and would not accumulate in the intestine. The adverse effects of Cd and polystyrene MPs on the intestinal microorganisms of broilers in the combined exposure group were reduced with the excretion of Cd and polystyrene MPs, which led to a decrease in LPS levels in the gut-liver axis, further reducing the expression level of the TLR4/MyD88/NF-κB inflammatory pathway, and also reducing the levels of various pro-inflammatory factors, thereby reducing inflammation of gut-liver axis. Therefore, although the combined exposure of polystyrene MPs and Cd still caused damage to the intestine and liver, the level of inflammation in the gut-liver axis was reduced.

Based on the above findings, it can be speculated that polystyrene MPs and Cd induce intestinal damage and liver inflammation in broilers by interfering with the TLR4/MyD88/NF-κB pathway and the homeostasis of intestinal flora, and the toxicity of combined exposure is lower than that of single exposure because of the adsorption of polystyrene MPs. This may provide a new idea for the treatment of polystyrene MPs or Cd poisoning. Due to the strong toxic effects of polystyrene MPs and Cd, it seems unrealistic to use one of the poisons to treat another poison in real life and clinical practice. Therefore, whether it is possible to find some new non-toxic substances that have no adverse effects on the body and can be adsorbed on polystyrene MPs (or adsorbed Cd) to be excreted is still worthy of further study.

## 5. Conclusions

MPs and Cd induced ileal inflammatory injury, intestinal physical, chemical and biological barrier damage, and liver inflammation in broilers by interfering with the TLR4/MyD88/NF-κB pathway and intestinal flora homeostasis, while the toxicity of combined exposure was lower than that of single exposure.

## Figures and Tables

**Table 1:** List of gene primers for qPCR.

Gene Name	Primer (5′–3′)	Sequence Number
*β-actin*	F:CCAGCCATGTATGTAGCCATCCAG R:GGTAACACCATCACCAGAGTCCATC	NM_205518.2
*IL-1β*	F:CAGAAGAAGCCTCGCCTGGATTC R:GCCTCCGCAGCAGTTTGGTC	NM_204524.2
*IL-2*	F:CGAGCTCTACACACCAACTGA R:ATCTTGCATTCACTTCCGGT	NM_204153.2
*IL-6*	F:AAATCCCTCCTCGCCAATCT R:CCCTCACGGTCTTCTCCATAAA	NM_204628.2
*TNF-α*	F:CCCAGTTCAGATGAGTTGCCCTTC R:GCCACCACACGACAGCCAAG	NM_204267.2
*IL-4*	F:CTTCCTCAACATGCGTCAGC R:TGAAGTAGTGTTGCCTGCTGC	NM_001007079.2
*IL-10*	F:CGGGAGCTGAGGGTGAA R:GTGAAGAAGCGGTGACAGC	NM_001004414.4
*MUC2*	F:GTGCCAGCAAACTTGTCGTT R:GCATTTGGAGATGGGCACAC	XM_040701656.2
*MUC5AC*	F:AAGACGGCATTTATTTCTCCAC R:TCATTACCAACAAGCCAGTGA	XM_052692775.1
*TLR4*	F:AGGCACCTGAGCTTTTCCTC R:TACCAACGTGAGGTTGAGCC	NM_001030693.2
*sIgA*	F:GTCACCGTCACCTGGACACCA R:ACCGATGGTCTCCTTCACATC	NM_204263.2
*Occludin*	F:CTGCTCTGCCTCATCTGCTTCTTC R:CCATCCGCCACGTTCTTCACC	NM_205128.1
*Claudin-1*	F:GACCAGGTGAAGAAGATGCGGATG R:CGAGCCACTCTGTTGCCATACC	NM_001013611.2
*ZO-1*	F:TCTTCCTCCTCCCGCTTCTTCAC R:AGAGATGGTGGTGTAGGCAGTGG	XM_040706827.2

## Data Availability

The data from this study are available upon reasonable request to the corresponding authors. The raw data of intestinal microbiota has been deposited into NCBI with BioProject ID: PRJNA1238088.
